# Endothelin‐1 mediates *Aspergillus fumigatus*‐induced airway inflammation and remodelling

**DOI:** 10.1111/cea.13367

**Published:** 2019-03-18

**Authors:** Briony Labram, Sara Namvar, Tracy Hussell, Sarah E. Herrick

**Affiliations:** ^1^ Division of Cell Matrix Biology and Regenerative Medicine Faculty of Biology Medicine and Health School of Biological Sciences University of Manchester Manchester UK; ^2^ Manchester Academic Health Science Centre Manchester UK; ^3^ Environment and Life Sciences University of Salford Greater Manchester UK; ^4^ Manchester Collaborative Centre for Inflammation Research (MCCIR) University of Manchester Manchester UK

**Keywords:** airway epithelium, *Aspergillus fumigatus*, asthma, endothelin‐1, remodelling

## Abstract

**Background:**

Asthma is a chronic inflammatory condition of the airways and patients sensitized to airborne fungi such as *Aspergillus fumigatus* have more severe asthma. Thickening of the bronchial subepithelial layer is a contributing factor to asthma severity for which no current treatment exists. Airway epithelium acts as an initial defence barrier to inhaled spores, orchestrating an inflammatory response and contributing to subepithelial fibrosis.

**Objective:**

We aimed to analyse the production of pro‐fibrogenic factors by airway epithelium in response to *A fumigatus*, in order to propose novel anti‐fibrotic strategies for fungal‐induced asthma.

**Methods:**

We assessed the induction of key pro‐fibrogenic factors, TGF‐β1, TGF‐β2, periostin and endothelin‐1, by human airway epithelial cells and in mice exposed to *A fumigatus* spores or secreted fungal factors.

**Results:**

*Aspergillus fumigatus* specifically caused production of endothelin‐1 by epithelial cells in vitro but not any of the other pro‐fibrogenic factors assessed. *A fumigatus* also induced endothelin‐1 in murine lungs, associated with extensive inflammation and airway remodelling. Using a selective endothelin‐1 receptor antagonist, we demonstrated for the first time that endothelin‐1 drives many features of airway remodelling and inflammation elicited by *A fumigatus*.

**Conclusion:**

Our findings are consistent with the hypothesis that elevated endothelin‐1 levels contribute to subepithelial thickening and highlight this factor as a possible therapeutic target for difficult‐to‐treat fungal‐induced asthma.

## 1 INTRODUCTION

Asthma is a chronic respiratory condition affecting approximately 300 million people world‐wide, accounting for around a quarter of a million deaths annually.^1^ Asthma is characterized by two main pathophysiological features, airway inflammation and airway remodelling, which together contribute to symptoms such as breathlessness, wheeze and persistent cough. Remodelling of the airways is poorly characterized, despite evidence suggesting its occurrence may proceed or occur in parallel to inflammation in childhood asthma.^2^, ^3^ Airway remodelling describes denuding of the epithelium, subepithelial fibrosis with increased extracellular matrix deposition, extensive smooth muscle hypertrophy and goblet cell hyperplasia.^4^, ^5^ Such airway remodelling contributes to the severity of exacerbations to aeroallergens such as those from house dust mite, pollen, animal dander and fungi. At present, no available therapy specifically targets the airway remodelling aspect of asthma.

Epidemiological studies have shown that severe asthma with fungal sensitization (SAFs) is associated with a high incidence of allergy to airborne fungi including *Aspergillus fumigatus (A fumigatus)*.^6^ It has been estimated that as many as 28% of people with asthma are hypersensitive to *A fumigatus*, but disease aetiology is unclear.^7^ *A fumigatus* spores can be found at high concentrations with the average adult inhaling several hundred per day.^8^ With a diameter of just 2‐3 μm, *A fumigatus* spores may disseminate throughout the airway reaching distal alveoli.^8^ In healthy individuals, inhaled spores are likely cleared by alveolar macrophages, but immunocompromised patients or those with reduced lung function are more prone to retain spores in their airway which may permit spore germination and prolonged host allergen exposure.^9^

Airway epithelium provides a physical barrier separating underlying tissue from the external environment and provides the first line of defence to inhaled *A fumigatus* spores. Through its pivotal role in recruiting innate immune cells,^10^ and mediating an adaptive immune response,^11^ airway epithelium is at the interface of host‐environment interactions and as such plays a significant role in regulating airway homeostasis.^12^, ^13^ Furthermore, signalling through an epithelial‐mesenchymal trophic unit (EMTU) may enable epithelial cells to regulate fibroblast behaviour in the subepithelial layer^14^ so governing the extent of repair following airway damage. Previous studies have shown that airway epithelial cells respond to germinating spores and hyphae of *A fumigatus* via production of a number of key cytokines including IL6, IL8, GM‐CSF and TNF‐α.^15^, ^16^ In addition, we and others have established that inhalation of components of A fumigatus in vivo elicits airway inflammatory and remodelling responses through release of secreted fungal products including allergens with protease activity.^17^, ^18^ However, it remains unclear whether *A fumigatus* spores and/or its components induce airway epithelium to produce pro‐fibrogenic growth factors, which may in turn contribute to airway remodelling and asthma severity.

Biopsies from asthmatic lungs show an up‐regulation of a number of pro‐fibrogenic factors including Transforming growth factor (TGF) β1 and β2, levels of which correlate with subepithelial fibrosis.^19^, ^20^ Periostin, a matricellular protein and promising asthma biomarker, is also up‐regulated in asthmatic airways and serum^21^, ^22^ and endothelin‐1 (ET‐1), an important contributor to organ fibrosis, is increased in exhaled breath condensate derived from people with asthma and in lavage fluid of atopic asthmatics.^23^, ^24^, ^25^ Furthermore, extensive evidence suggests a role for ET‐1 in remodelling and fibrosis of the airway associated with bronchiectasis, idiopathic pulmonary fibrosis and scleroderma lung disease, suggesting that this growth factor may be central for driving lung fibrosis in multiple settings.^26^ These growth factors have been shown to elicit fibrogenic effects in cultured fibroblasts^27^, ^28^, ^29^ and contribute to airway remodelling events in vivo following exposure to aeroallergens such as house dust mite extract and ovalbumin.^30^, ^31^, ^32^ However, the major pro‐fibrogenic growth factors likely to contribute to *A fumigatus*‐induced airway remodelling have not yet been defined. The purpose of this study was to elucidate the growth factors produced following *A fumigatus* inhalation that drive subepithelial fibrosis in order to identify therapeutic targets.

## 2 METHODS

### 2.1 *Aspergillus fumigatus* culture

*Aspergillus fumigatus* strain Af293 was used, originally obtained from Manchester mycology reference centre (Wythenshawe, UK) and kindly gifted by P. Bowyer (University of Manchester). *A fumigatus* was cultured on Sabouraud dextrose agar (Oxoid, Hampshire, UK) at 37°C for 5 days. Spores were harvested with a vigorous PBS Tween (0.05% tween 20) wash and hyphae removed using sterilized lens cloth. For in vivo studies, spores were harvested as described with a minor modification of using 0.05% tween 80. Spores were then passed through 40 μm nylon mesh and centrifuged for 5 minutes at 10 000 *g* at 4°C twice. The concentration of spores was adjusted to 5 × 10^8^ spores/mL, aliquoted and frozen. Culture filtrates were produced according to our previously described protocol.^18^ Briefly, Erlenmeyer flasks containing 500 mL Vogel's minimal media were inoculated with 500 × 10^6^ spores/mL and cultured for 48 hours at 37°C at 320 rpm. Resultant cultures were filtered through J cloth and sterile filtered (0.2 μm). Filtrates were dialysed overnight, freeze‐dried and stored at −80°C. Freeze‐dried aliquots were reconstituted with sterile PBS, and total protein content was determined using the BCA protein assay before use (Thermo Scientific, Loughborough, UK).

### 2.2 Bronchial epithelial cell culture and exposure to *A fumigatus*

Human primary bronchial epithelial cells (BECs) were purchased from Promocell (Heidelberg, Germany) and Lonza (Basel, Switzerland). Cells were cultured in Bronchial Epithelial Cell Growth Media supplemented with BEGM BulletKit (Lonza) in 75‐cm^2^ flasks until they reached 80% confluence. For experiments, BECs were used between passages 2 and 3 and seeded at 15 × 10^3^/cm^2^. Monolayers were exposed to 1 × 10^5^ spores/mL for 12 and 24 hours or 1 μg/mL *A fumigatus* culture filtrate for 24 hours. At the end of the study, culture supernatants were collected and levels of TGF‐β1, ET‐1, periostin and TGF‐β2 determined using DuoSet^®^ ELISA kits performed according to manufacturer's instructions (R&D Systems, Abingdon, UK). For cultures involving germinating spores, cell layers were collected for analysis of gene expression, whilst supernatants were filtered through a 0.22 μm filter for ELISA.

In some experiments, in order to assess the growth of *A fumigatus* in the presence of epithelial cells, cultures were stained for calcofluor‐white (Sigma‐Aldrich, Poole, UK) and time‐lapse imaging performed using the Nikon Eclipse TE2000E microscope at X20 using an ORCA‐ER CCD camera (Hamamatsu, Welwyn Garden City, UK).

### 2.3 Murine models of *A fumigatus* induced airway inflammation and remodelling

**Figure 1:**
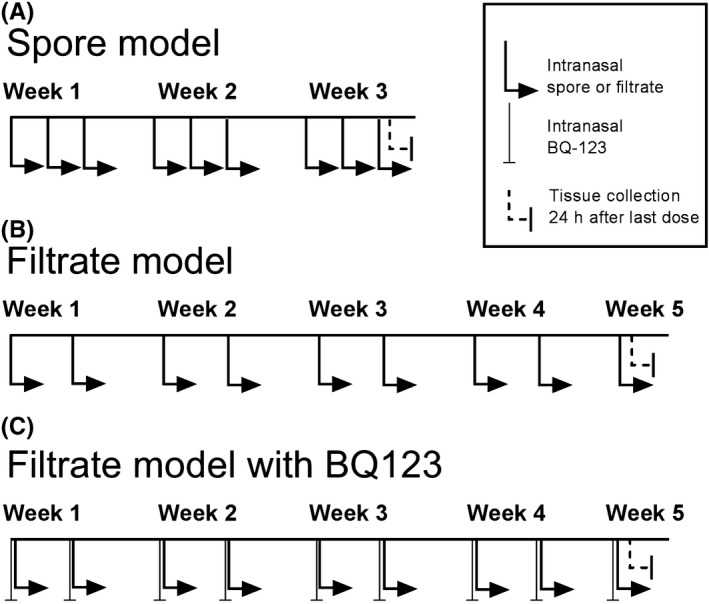
Schematic representation of the experimental design of the murine models of *Aspergillus fumigatus* exposure. A, Spore inhalation model involved mice receiving an intranasal dose of spores nine times over three consecutive weeks, and sample collection performed 24 h after final exposure. B, Culture filtrate model involved mice receiving an intranasal dose of culture filtrate nine times over five consecutive weeks with sample collection performed 24 h after final exposure. C, A separate group of mice also received an intranasal dose of BQ‐123, an ET‐1 receptor antagonist, 30 min prior to *A fumigatus* culture filtrate exposure

Male C57BL/6J mice, aged 8 weeks (Charles River Laboratories, Harlow, UK), were maintained under specific pathogen‐free conditions for the duration of the study with food and water available ad libitum. All procedures were performed in accordance with the UK Animal Scientific Procedures Act 1986 with local ethical committee approval. For the *A fumigatus* spore exposure model, mice were anesthetized with 2%‐3% isoflurane and 40 μL of 4 × 10^5^ spores in PBS Tween 80 (0.05%) or PBS Tween 80 (0.05%) alone was administered intranasally (Figure 1A). Mice were dosed a total of nine times over three consecutive weeks following a previously published protocol.^33^ For the *A fumigatus* culture filtrate exposure model, mice were anesthetized with 2%‐3% isoflurane and 25 μL of the culture filtrate (containing 50 μg of protein) or PBS was administered intranasally (Figure 1B). Mice were dosed twice a week for 4 weeks followed by a final dose on week five following our previous protocol.^18^ In studies, involving ET‐1 receptor antagonist (BQ‐123; Sigma), 50 pmol of antagonist in 25 μL PBS or PBS alone was intranasally dosed 30 minutes prior to culture filtrate administration (Figure 1C). Twenty‐four hours after final *A fumigatus* exposure, animals were killed and samples including bronchoalveolar lavage fluid (BALF), serum and lung collected, processed and analysed as previously described.^18^ Cytokines and growth factors were assessed in BALF and lung homogenate and IgE in serum by ELISA (Data S1).

### 2.4 Real‐time PCR for growth factor gene expression

RNA was extracted from cell monolayers and frozen homogenized lung samples using the RNeasy Mini Kit (Qiagen, Crawley, UK). Reverse transcription was performed using TaqMan Reverse Transcription Reagents (ThermoFisher Scientific). Using the SensiFAST SYBR No‐ROX Kit (Bioline, London, UK), qRT‐PCR reactions were performed in technical triplicate using forward and reverse primers for gene expression of ET‐1, TGF‐β1, TGF‐β2, periostin and normalized to GAPDH or RPL13 as housekeeping genes (Data S1).

### 2.5 Histology and immunofluorescence

The entire left lobe was fixed in buffered paraformaldehyde and wax embedded to permit direct comparison between experimental animals. Serial transverse 8 μm lung sections from the same region in each lung were stained with haematoxylin and eosin (H&E) or Masson's trichrome. For analysis of subepithelial collagen thickness, at least five images of bronchioles within ×20 magnification field of view, from the same region of the lung were captured for each animal. For immunofluorescence, sections were permeabilized and then incubated with primary antibody to α‐SMA (A2547—clone 1A4; Sigma‐Aldrich) diluted at 1:400, washed and then followed by secondary antibody (Alexa Fluor 488 goat anti‐mouse IgG, 1:1000; Life Technologies, Oregon, USA) before mounting (Vectashield with DAPI, Cambridgeshire, UK). For analysis of α‐SMA immunostaining, images of bronchioles that were of appropriate size to be contained within fields of view under high‐power magnification (×20) were obtained from the same region of the lung using a ZEISS Axiostar plus microscope. For image analysis, collagen staining from Masson's trichrome‐stained images was isolated by colour deconvolution. Derived images from colour deconvolution were made binary, and the total area of the bronchiole and subepithelial region showing positive α‐SMA or Masson's staining was manually selected. The percentage area with positive stain was then determined by Image J Analysis Software (National Institute of Health, Maryland, USA). A minimum of five bronchioles were used from each lung for analysis.

### 2.6 Statistical analysis

Statistical analysis was performed using GraphPad Prism 6 for Windows (GraphPad Software Inc, California, USA) using one‐way ANOVA with post hoc tests or Student's *t* tests as appropriate. Observed change was considered significant with *P* < 0.05. Data are presented as mean ± SEM.

## 3 RESULTS

### 3.1 Bronchial epithelial cells up‐regulate Endothelin‐1 expression in response to *A fumigatus* spores

**Figure 2:**
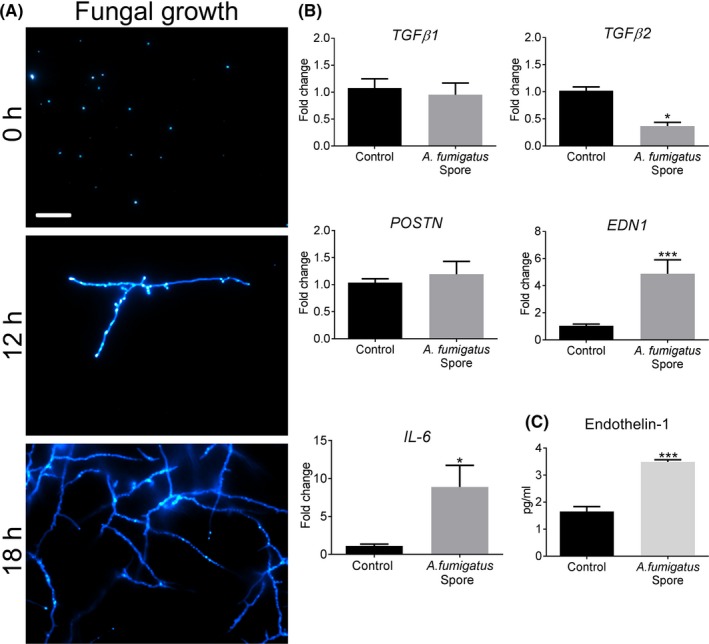
ET‐1 is up‐regulated in human bronchial epithelial cells exposed to *Aspergillus fumigatus* germinating spores. A, Confocal microscopy of live, germinating spores seeded onto BEC monolayers and stained with calcofluor‐white at 0, 12 and 18 h. Note the progressive emergence of hyphal extensions (Scale bar = 100 μm). B, In response to *A fumigatus* germinating spore exposure for 12 h, BECs increased gene expression of *EDN1* (***P < 0.001, n = 6) and pro‐inflammatory mediator, *IL‐6* (*P ≤ 0.05, n = 6) as assessed by qPCR. Gene expression of other pro‐fibrogenic mediators, *TGF‐β1* and *POSTN*, was unchanged whilst *TGF‐β2* expression was significantly reduced (*P < 0.05, n = 6) relative to control. C, In response to *A fumigatus* germinating spores, BECs significantly increased the production of ET‐1 after 24 h (***P < 0.001, n = 6)

We initially determined whether *A fumigatus* induced human BECs to express pro‐fibrogenic growth factors in vitro. Cells were exposed to spores and expression of TGF‐β1 and TGF‐β2, periostin and ET‐1 assessed by qPCR. At 12 hours, *A fumigatus* spores had undergone germination, showing progressive branching of hyphae which gradually evolved into a mycelial mesh by 18 hours (Figure 2A). In response to *A fumigatus* spores, there was no increase in gene expression for TGF‐β1 or periostin and surprisingly a down‐regulation of TGF‐β2 by BECs (Figure 2B). In contrast, *A fumigatus* spores caused a highly significant increase in ET‐1 gene expression and the pro‐inflammatory cytokine, IL6 (Figure 2B). Furthermore, in response to *A fumigatus*, ET‐1 protein production was significantly increased at 24 hours compared with control (Figure 2C). In parallel, BECs were exposed to *A fumigatus* culture filtrate containing secreted products, and again, there was a significant increase in gene expression and protein production of ET‐1 but not of the other growth factors assessed (Figure S1).

### 3.2 Induction of Endothelin‐1 in a murine *A fumigatus* spore inhalation model

**Figure 3:**
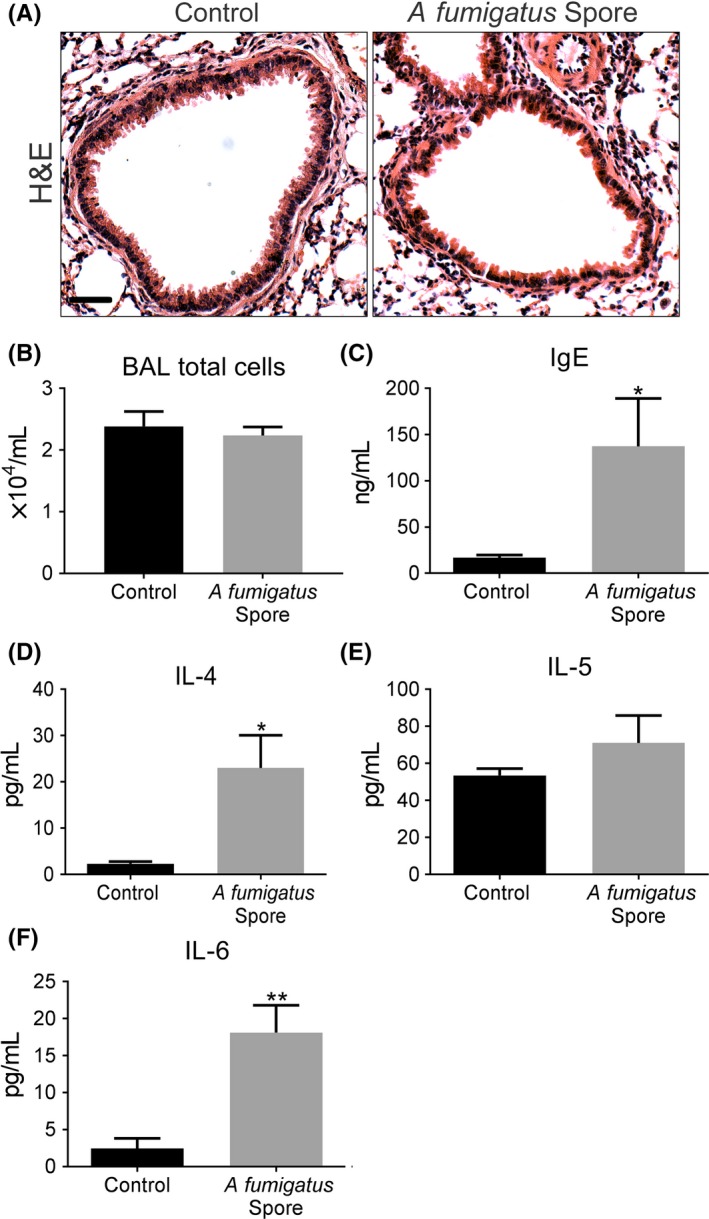
*Aspergillus fumigatus* spores elicit a mild inflammatory and allergic response in a murine inhalation model. A, Representative H&E images, depicting the relatively mild peribronchiolar inflammatory response in airways exposed to *A fumigatus* spores (Scale bar = 50 μm). B, Total BAL cell counts were similar in response to spore exposure and control. C‐F, Exposure to spores caused a mild, but a significant increase in serum IgE (*P < 0.05, n = 5) and IL4 (*P < 0.05, n = 5) and IL6 (**P < 0.01, n = 5) levels in homogenized lung, but no change for IL5 compared with control

**Figure 4:**
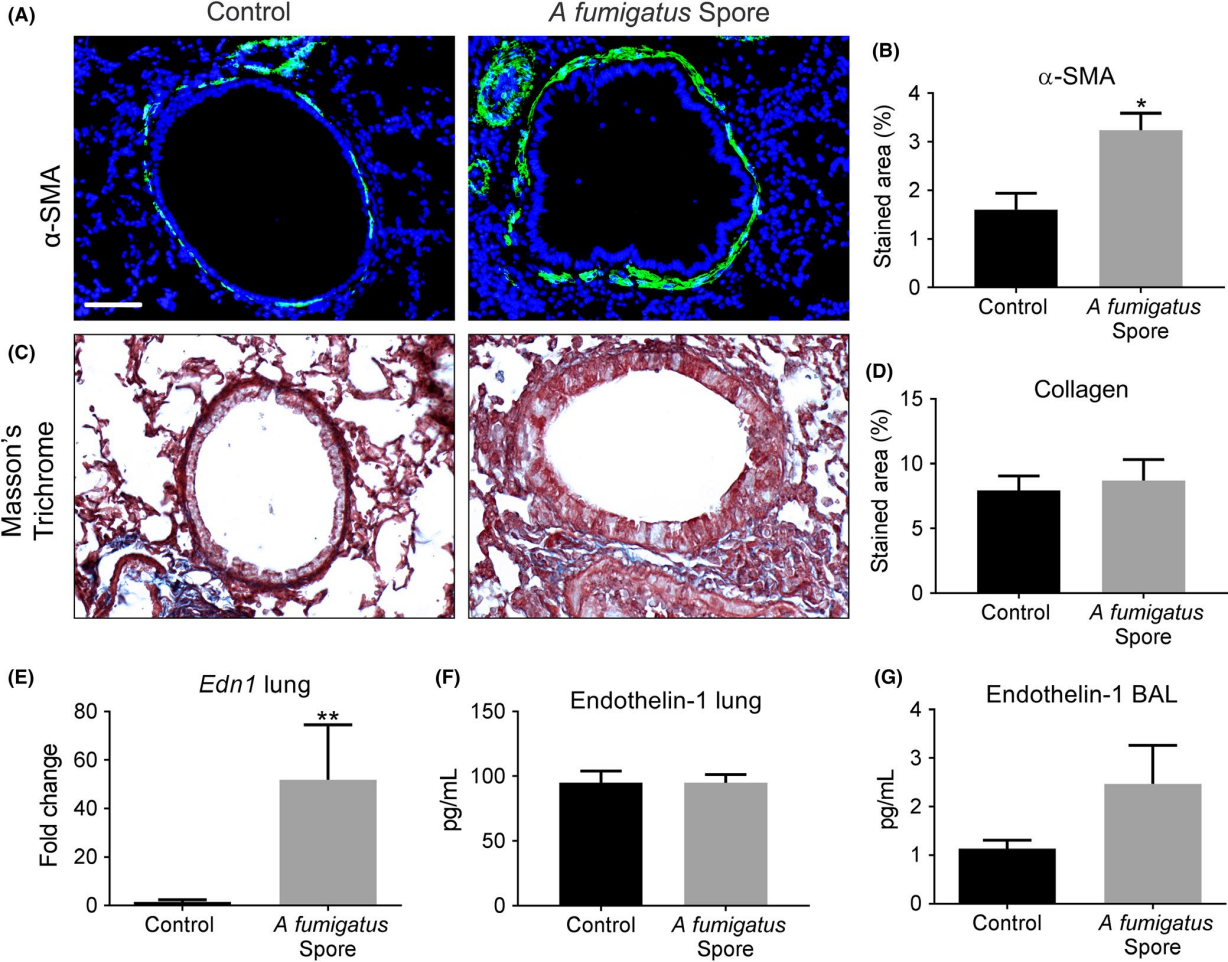
*Aspergillus fumigatus* spores cause limited remodelling of the airways and Endothelin‐1 induction in a murine inhalation model. A‐B, Repeated exposure to *A fumigatus* spores significantly increased peribronchiolar α‐SMA (green and counterstained for DAPI to visualize nuclei blue; *P < 0.05; n = 5) compared with control (Scale bar = 50 μm). C‐D, No detectable change in collagen deposition around airways following spore exposure was detected by image analysis of Masson's trichrome‐stained sections. E‐G, A significant increase in lung *Edn1* gene expression (**P < 0.01, n = 5) was found by qPCR in spore exposed mice but no significant increase in ET‐1 protein in total lung homogenate or BAL compared with control mice

Using a murine model of repeated spore inhalation (Figure 1A), we next analysed the ability of *A fumigatus* to up‐regulate pro‐fibrogenic growth factors in vivo. Mouse airway exposure to *A fumigatus* spores, over the course of 3 weeks, was associated with a mild inflammatory response of the peribronchiolar region (Figure 3A) with no significant difference in total cell count in BAL compared with control (Figure 3B). Exposure to spores was also associated with a relatively mild, but significant increase in the level of pro‐inflammatory cytokines, IL4 and IL6, assessed in lung homogenate, as well as a significant rise in total serum IgE but not IL5 (Figures 3C‐F). *A fumigatus* spore exposure also significantly enhanced α‐SMA localization around the airways (Figure 4A‐B), although no significant change in peribronchiolar collagen deposition was detected by image analysis (Figure 4C‐D). This relatively mild remodelling of the airways in response to *A fumigatus* spores was accompanied by significantly increased lung ET‐1 gene expression (Figure 4E). However, the increase in gene expression was not accompanied by a significant increase in ET‐1 protein level in lung homogenate or BAL compared with controls (Figure 4F‐G). Similar to findings in vitro, *A fumigatus* spores failed to induce a significant up‐regulation of gene expression for TGF‐β1, TGF‐β2 and periostin in murine lung tissue (Figure S2). Together, these findings indicate that airway ET‐1 gene expression was specifically up‐regulated in response to *A fumigatus* spores.

### 3.3 *Aspergillus fumigatus* culture filtrate drives robust airway inflammation and remodelling associated with Endothelin‐1 induction

**Figure 5:**
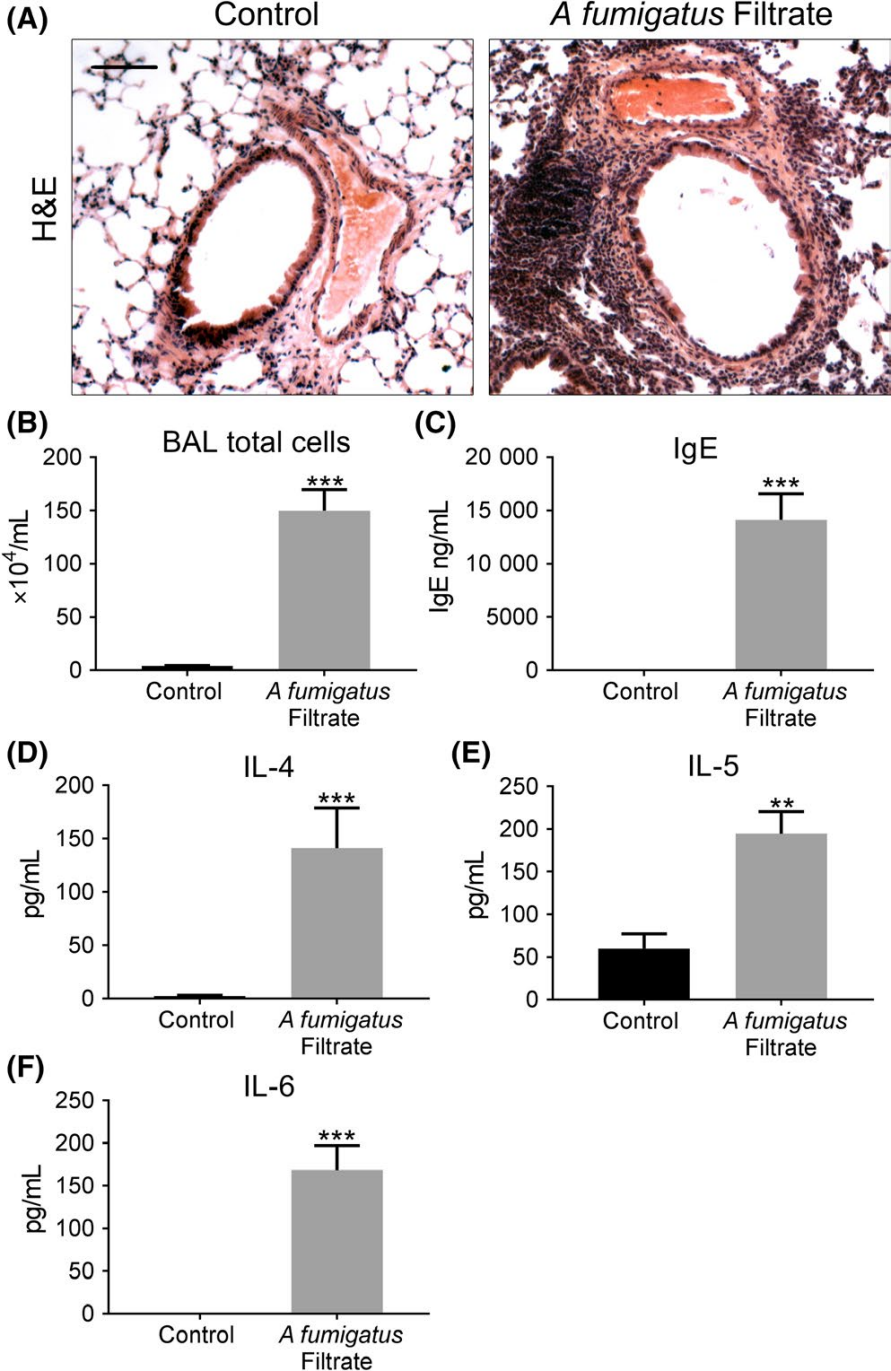
Robust inflammation and allergic response in a murine *A fumigatus* culture filtrate inhalation model. A, Representative H&E images of control and *A fumigatus* culture filtrate exposed airways (Scale bar = 50 μm). Note the profound peribronchiolar and perivascular inflammation apparent in culture filtrate exposed airways. B, Total cell counts from Giemsa‐stained cytospins showing a significant increase in total cell number (***P < 0.001, n = 5), C‐F, Total serum IgE (***P < 0.001, n = 5) and pro‐inflammatory and Th2‐promoting cytokines, IL4 (***P < 0.001, n = 5), IL‐5 (**P < 0.01, n = 5) and IL‐6 (***P < 0.001, n = 5) were all significantly increased in the lungs of mice exposed to culture filtrate compared with control

**Figure 6:**
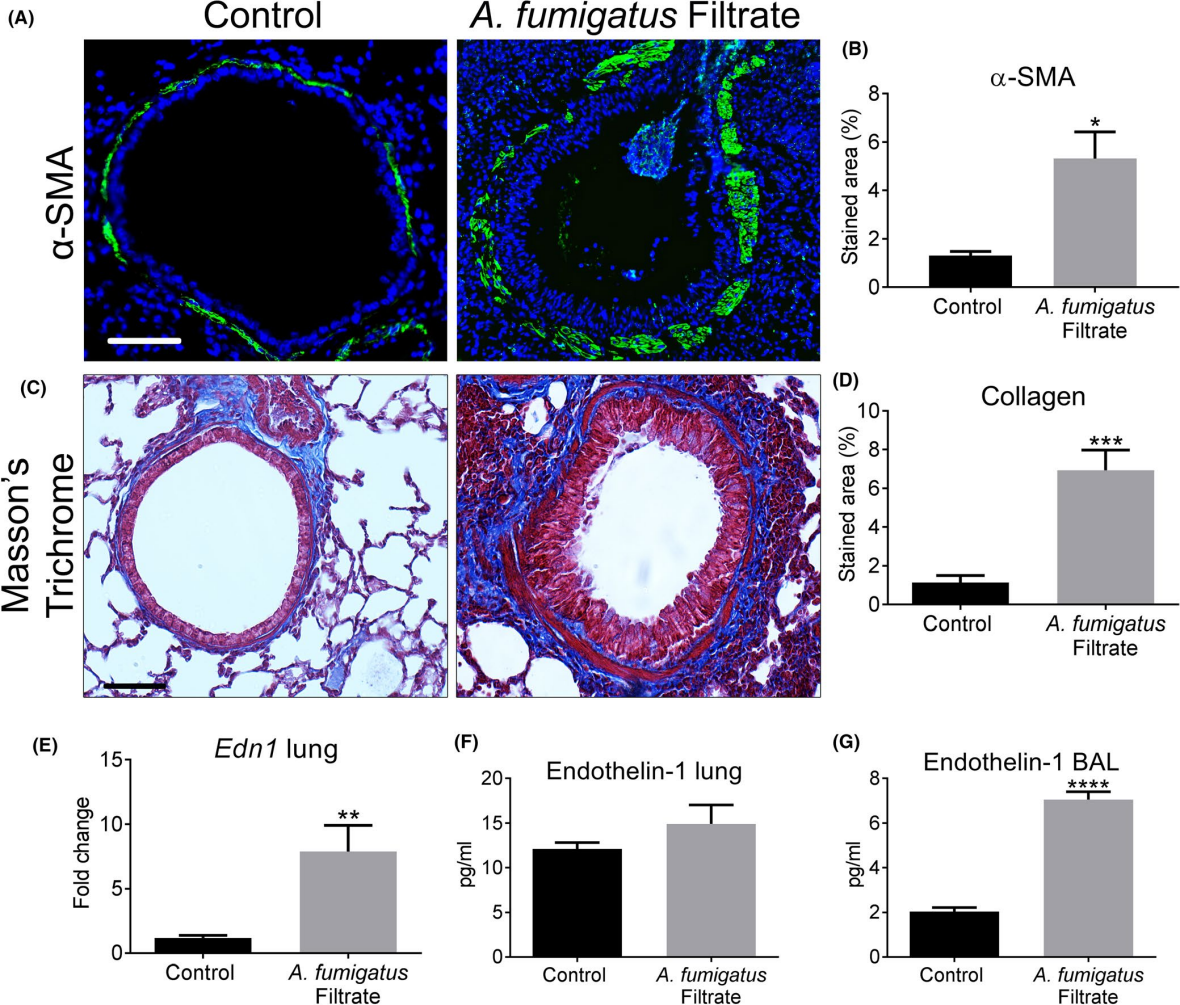
Extensive airway remodelling in mice exposed to *Aspergillus fumigatus* culture filtrate. A‐B, Culture filtrate caused a noticeable increase in peribronchiolar α‐SMA localization (green and counterstained for DAPI to visualize nuclei, blue). compared with control which was found to be significant following image analysis (*P < 0.05, n = 5). C‐D, Culture filtrate exposed bronchioles showed extensive collagen deposition on Masson's trichrome‐stained sections confirmed to be significantly increased compared with control by image analysis (***P < 0.001, n = 5; Scale bar = 50 μm). This profound airway wall remodelling in culture filtrate exposed mice was associated with a significant increase in E, *Edn1* gene expression in homogenized lung (**P < 0.001, n = 5), F, no change in total lung homogenate ET‐1 protein, but G, a robust increase in ET‐1 protein in BAL (****P < 0.0001, n = 5) compared with control

We conceptualized that rapid fungal spore clearance before adequate germination in the inhalation model may prevent sufficient exposure time of the airways to *A fumigatus* mediators. We therefore used a different inhalation model which involved repeated airway exposure to *A fumigatus* culture filtrate in vivo over the course of a 5‐week period (Figure 1B). Prominent peribronchiolar inflammation was evident in culture filtrate exposed lungs (Figure 5A) associated with a significant increase in total cell counts in BAL compared with that from control lungs (Figure 5B). Differential BAL cell counts revealed that this overall increase was associated with a decrease in the number of macrophages concomitant with an increase in the number of eosinophils, neutrophils and lymphocytes (Figure S3). Filtrate‐induced inflammation was associated with a robust and highly significant increase in pro‐inflammatory cytokines, IL4, IL5 and IL6, in lung homogenate and total serum IgE to levels far greater than that found in response to spores indicating a robust allergic response (Figure 5C‐F). Furthermore, significantly increased α‐SMA localization was detected around the airways accompanied by profound collagen deposition, hallmarks of airway remodelling (Figure 6A‐D). Gene expression of lung ET‐1 was significantly increased in culture filtrate exposed lungs (Figure 6E). Similar to the spore model, lung homogenate ET‐1 protein was not changed (Figure 6F), but interestingly, ET‐1 levels in BAL were significantly increased which may suggest an increase in bronchial epithelial‐derived ET‐1 or increased production by inflammatory cells in BAL (Figure 6G). We also assessed gene expression of TGF‐β1, TGF‐β2 and periostin in the lungs of mice exposed to *A fumigatus* culture filtrate. Similar to the spore inhalation model, culture filtrate exposure did not increase the expression of these growth factors (Figure S4).

### 3.4 Endothelin receptor A (ET_A_) antagonism diminishes *A fumigatus*‐induced airway pathology

**Figure 7:**
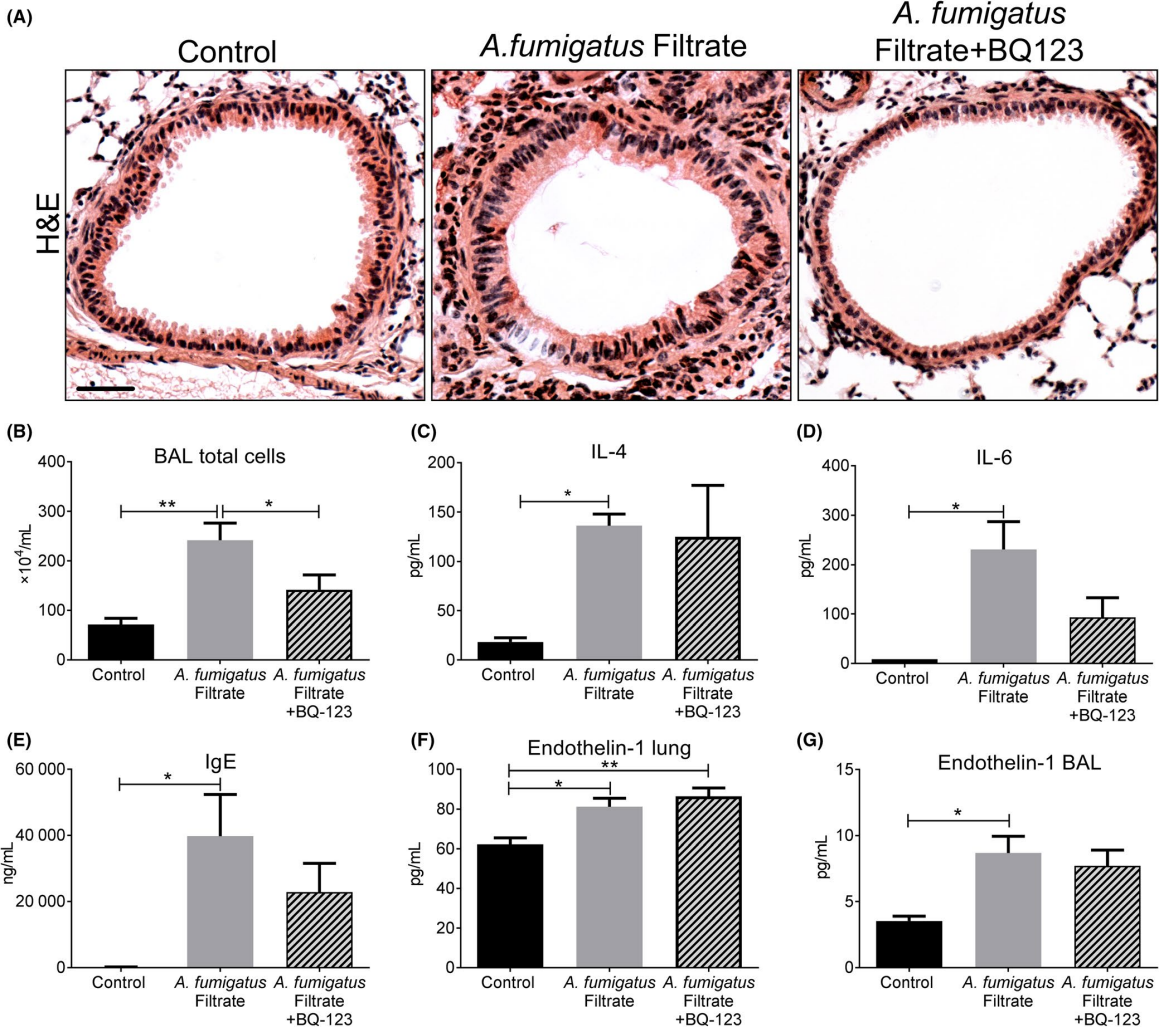
Endothelin‐1 receptor antagonism moderates inflammation and allergic response to *Aspergillus fumigatus*. A, Representative H&E images showing exposure to *A fumigatus* culture filtrate caused extensive peribronchiolar inflammation which was far less apparent following BQ‐123 treatment (Scale bar = 50 μm). B, Mice receiving culture filtrate alone showed a significant increase in total BAL cell count (**P < 0.01, n = 5) which was significantly reduced by BQ‐123 treatment relative to culture filtrate group (*P < 0.05, n = 5). C, Exposure to culture filtrate caused a significant induction of IL4 (*P < 0.05, n = 5), which was unchanged with BQ‐123 treatment and D, a significant induction of IL6 (**P < 0.01, n = 5) that was decreased by BQ‐123 abet not significantly. E, Culture filtrate caused a significant induction of total serum IgE (*P < 0.05, n = 5) which also showed a trend for reduction following BQ‐123 treatment. F, *A fumigatus* caused a significant induction of ET‐1 protein in the lung (*P < 0.05, n = 5), which increased further with BQ‐123 treatment (**P < 0.01, n = 5). G, Relative to controls, ET‐1 was significantly increased in BAL from mice exposed to culture filtrate (*P < 0.05, n = 5) and did not statistically significant change in the BQ‐123‐treated group

**Figure 8:**
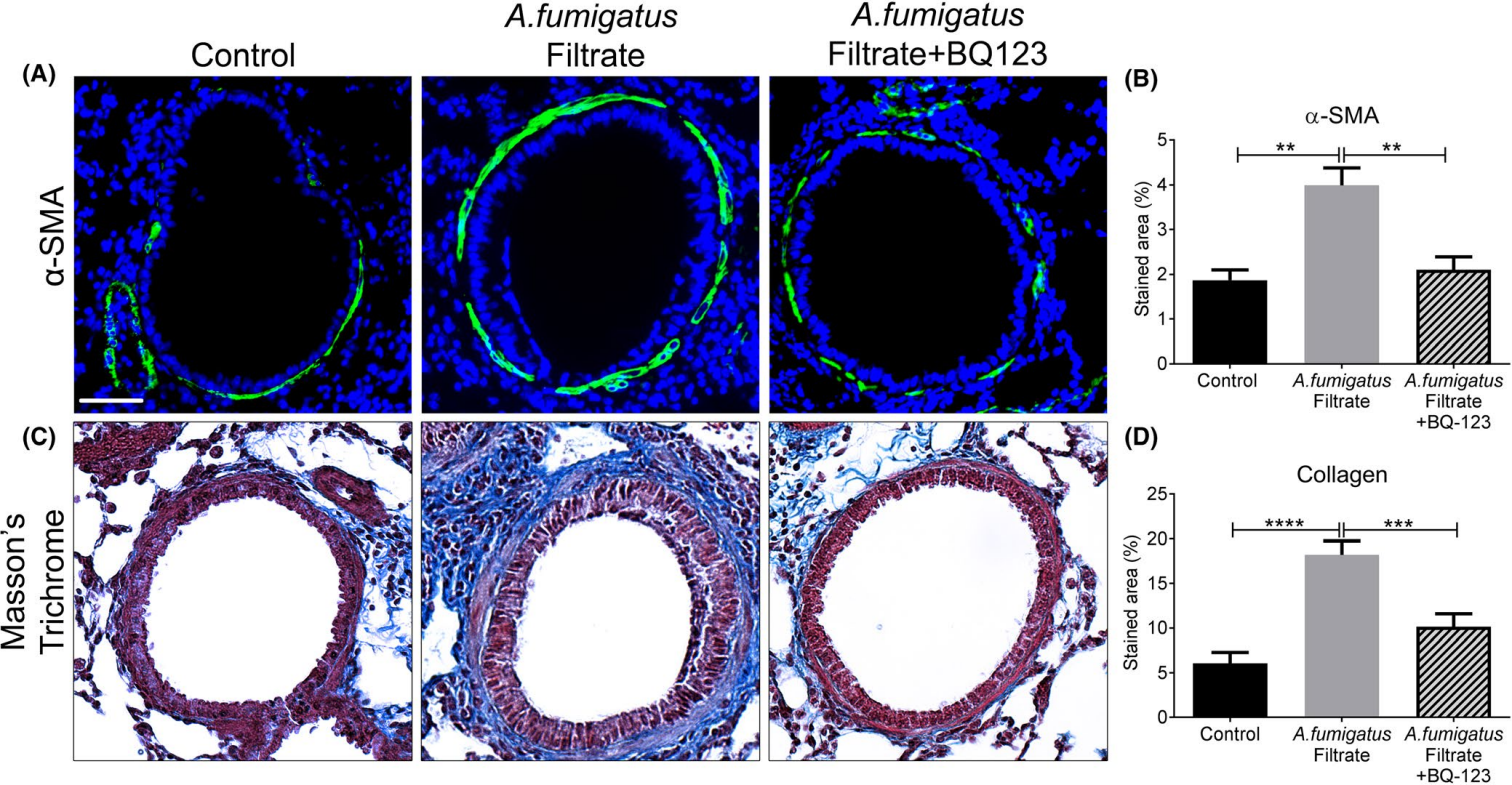
Endothelin‐1 receptor antagonism obliterates *Aspergillus fumigatus*‐induced airway wall remodelling. A, Representative images of bronchioles from control, culture filtrate exposed or culture filtrate with BQ‐123 pre‐treatment mice showing α‐SMA localization (green) and counterstained for DAPI to visualize nuclei (blue). B, Compared with controls, *A fumigatus* filtrate caused a significant increase in peribronchiolar α‐SMA (**P < 0.01, n = 5) which was significantly decreased in the *A fumigatus* filtrate + BQ‐123 group compared with culture filtrate group (**P < 0.01, n = 5). C, Representative images of bronchioles from control, *A fumigatus* filtrate or *A fumigatus* filtrate + BQ‐123‐treated mice stained by Masson's trichrome. D, Exposure to *A fumigatus* filtrate caused a profound and significant increase in collagen (****P < 0.0001, n = 5) which was significantly diminished in the BQ‐123‐treated group (***P < 0.001, n = 5)

We hypothesized that ET‐1 likely facilitates *A fumigatus* driven airway pathology. To test this theory, mice were treated intranasally with BQ‐123, an ET_A_ receptor antagonist, prior to each *A fumigatus* culture filtrate exposure. Pre‐treatment with BQ‐123 reduced the extent of peribronchiolar inflammatory infiltration and significantly reduced total BAL cell count compared to mice receiving filtrate alone (Figure 7A‐B). Assessment of BAL differential cell counts revealed that this reduction was due to a significant decrease in the number of macrophages, neutrophils and lymphocytes (Figure S5). Reduced inflammation was not associated with a significant reduction in IL4, IL6 or total serum IgE in the BQ‐123‐treated group compared with culture filtrate alone group (Figure 7C‐E). Antagonism of ET‐1 receptor caused a modest, but significant increase in lung homogenate ET‐1, but did not alter BAL ET‐1 levels compared with mice receiving culture filtrate (Figure 7F‐G). We next assessed whether BQ‐123 reduced airway remodelling in response to *A fumigatus* culture filtrate. Subepithelial α‐SMA distribution (Figure 8A‐B) and collagen deposition (Figure 8C‐D) induced by culture filtrate exposure were both significantly diminished and often undetectable in the airways of mice pre‐treated with BQ‐123. These findings suggest that ET_A_ antagonism successfully diminishes the inflammatory response and subepithelial remodelling induced by *A fumigatus* secreted products.

## 4 DISCUSSION

In the present study, we demonstrated that *A fumigatus* spores and culture filtrate caused a highly specific up‐regulation of ET‐1 in cultured human airway epithelial cells. By modelling fungal‐induced allergic disease in mice, we corroborated these findings in vivo and showed that *A fumigatus* driven airway inflammation and remodelling was associated with a targeted up‐regulation of ET‐1. Based on the notion that ET‐1 is central to *A fumigatus* driven airway remodelling, we delivered an ET receptor A (ET_A_) antagonist, BQ‐123, prior to exposing mice to *A fumigatus*. We demonstrated for the first time that antagonism of ET_A_ prevents *A fumigatus*‐induced inflammation and remodelling of the airways.

Previously regarded as a mere bystander, the airway epithelium is now recognized as pivotal in driving the asthma phenotype.^10^, ^11^, ^12^ Furthermore, acting as an epithelial‐mesenchymal trophic unit, injured airway epithelial cells signal to underlying mesenchymal cells and vice versa.^14^ *A fumigatus* up‐regulates a number of key cytokines in airway epithelial cells.^15^, ^16^ Intriguingly in the present study, we found that both *A fumigatus* spores and culture filtrate also caused an up‐regulation of ET‐1 in airway epithelial cells, with no significant change detected for TGF‐β1 or periostin and a decrease in TGF‐β2 compared with untreated controls. We next substantiated the up‐regulation of ET‐1 found in vitro using murine models of allergic inflammation mediated by *A fumigatus* exposure. Both a spore inhalation model^33^ and our previously published model of culture filtrate exposure^18^ showed a significant up‐regulation of ET‐1 gene expression compared with controls. In accord with our in vitro findings, TGF‐β1 and β2 and periostin expressions were not significantly altered. Up‐regulation of ET‐1 expression was accompanied by Th2 cytokine and IgE induction and extensive remodelling of the airways, with subepithelial collagen deposition and smooth muscle hypertrophy, highly pronounced in the culture filtrate model. To our knowledge, this is the first report of an up‐regulation of ET‐1 by human airway epithelial cells and in BAL from murine lungs exposed by *A fumigatus*. These findings may indicate an epithelial source of this growth factor in vivo. ET‐1 has been found in high levels in children with asthma^24^ and also increased during exacerbation of asthma in adults.^25^ Furthermore in human asthmatic airways, ET‐1 is located primarily in the bronchial epithelium^34^ with its expression increased in steroid‐refractory asthma.^35^ As well as the epithelium, ET‐1 is produced by a number of lung cell types including pulmonary vascular endothelial cells, macrophages, neutrophils and fibroblasts.^36^, ^37^, ^38^, ^39^ ET‐1 is also reported to drive macrophage cytokine production and recruitment of lymphocytes, neutrophils and eosinophils in ovalbumin‐sensitized mice.^40^ In the current study, macrophages, neutrophils and lymphocytes were all increased in the BAL of *A fumigatus*‐exposed mice. It is therefore possible that ET‐1 was derived from the bronchial epithelium and contributes to the recruitment of immune cells found in BAL and/or was produced by these immune cells.

ET‐1 signals via ET receptor A (ET_A_), expressed by many cell types including vascular and airway smooth muscle leading to vaso‐ and bronchoconstriction but can also signal by ET receptor B (ET_B_), predominately expressed by the endothelium.^41^ In the current study, up‐regulation of ET‐1 in mice exposed to *A fumigatus* culture filtrate was associated with an increased inflammatory response. Treatment with ET_A_ antagonist, BQ‐123, diminished the recruitment of inflammatory cells around the airways and total cell counts assessed in lung lavage. Differential cell counts showed that this was due to a significant decline in the number of macrophages, neutrophils and lymphocytes following BQ‐123 treatment. A range of immune cells including macrophages, dendritic cells and lymphocytes also express ET receptors,^42^ providing the signalling mechanism by which up‐regulated ET‐1 may drive early inflammation and ultimately an allergic phenotype. It may also be the means by which BQ‐123 diminished inflammation and allergy. These finding support those of others where inhibition of ET‐1 with BQ‐123 and a dual ET_A_ and ET_B_ blockade with SB‐209670, reduced airway eosinophilia and neutrophilia in ovalbumin‐sensitized mice.^43^ Furthermore, in a mouse model of house dust mite sensitization, eosinophilia and airway hyperresponsiveness were alleviated by the dual ET‐1 receptor antagonist SB‐217242.^44^ Interestingly, eosinophil cell count was not significantly reduced by BQ‐123 in the current study although there was only a modest increase with culture filtrate exposure. Overall, our findings point to an important pathophysiological role for ET‐1 in the development of airway inflammation in *A fumigatus*‐induced allergic asthma.

As well as mediating an inflammatory response, our in vivo findings support a role for *A fumigatus*‐induced ET‐1 in mediating subepithelial fibrosis. Antagonism of ET_A_ with BQ‐123 caused near‐complete resolution of *A fumigatus*‐induced subepithelial collagen deposition and diminished α‐SMA‐positive immunostaining around airways. Previous in vitro studies showed that ET‐1 elicits fibroblast proliferation, differentiation into myofibroblasts and induction of contractile activity.^36^, ^45^, ^46^, ^47^ Intriguingly, ET_A_ antagonism also inhibited the differentiation of isolated blood‐derived fibrocytes into myofibroblasts in vitro.^48^ Furthermore, adenovirus‐mediated pulmonary up‐regulation of ET‐1 was sufficient to drive extensive inflammation coupled with remodelling of the airways.^49^ Of relevance, a study involving ovalbumin exposure in mice overexpressing *smad 2*, a downstream TGF‐β signalling molecule, displayed reduced airway wall remodelling following ET‐1 antagonism.^50^ Lastly, cultured bronchial epithelial cells were shown to display reduced migration and proliferation in the presence of ET‐1, suggesting this factor could potentially lead to defective repair of the lung epithelium resulting in enhanced remodelling.^51^ Taken together, such data point to a possible role for ET‐1 in the epithelial‐mesenchymal trophic unit, where *A fumigatus*‐induced activation of airway epithelium may trigger the production of ET‐1 that initiates a fibrogenic response in the subepithelial layer. These experimental observations are interesting when considering the pathophysiology of childhood asthma, where remodelling of the airways may occur in parallel or precede inflammation.^2^ Our findings build on these previous reports and show that antagonizing ET_A_ is an effective treatment to combat both inflammation and remodelling caused by inhaled fungal particles. This finding is particularly significant for difficult‐to‐treat asthma patients, quite often sensitized to airborne fungi.

In our hands, the extent of airway inflammation and remodelling was relatively mild in the *A fumigatus* spore inhalation model. This may stem from rapid spore clearance by innate immune cells recruited to the airways, ultimately not providing sufficient time for complete spore germination and host sensitization.^52^ Shedding of the outer rodlet layer and exposure of carbohydrate moieties during germination are thought to be crucial steps in the host inflammatory response.^53^ Indeed, studies comparing repeated exposure to live or dead *A fumigatus* spores in pre‐sensitized mice have shown that germination is essential for allergic airway inflammation and remodelling.^54^ We assessed fungal burden 24 hours after final spore exposure and found no evidence of *A fumigatus* colonization of the lungs supporting this conclusion (data not shown). Repeated exposure to a high concentration of secreted fungal factors, as provided by the culture filtrate, was much more efficient in driving inflammation and airway remodelling than that found with the rapidly cleared spores.

It remains uncertain which secreted mediators from germinating spores and enriched in fungal culture filtrate may induce ET‐1 production but could include fungal protease allergens and/or secondary metabolic by‐products. We previously showed that deletion of specific protease activity from the culture filtrate of a genetically modified *A fumigatus* isolate curtailed epithelial damage and airway remodelling in the mouse inhalation model.^18^ Others have shown that proteases contained in various aeroallergens such as ragweed, cockroach and house dust mite, can activate PAR2, a seven‐transmembrane G‐coupled protein receptor. Furthermore, *A fumigatus* extract has been shown to activate this receptor in airway epithelial cells and biases the cells to mediate a Th2 response.^55^ Of note, activation of this receptor in keratinocytes stimulated by house dust mite‐derived proteases increased ET‐1 production in vitro. Therefore, activation of PAR2 by *A fumigatus* proteases may be a proposed mechanism leading to ET‐1 induction. However, in the current study, we used culture filtrate derived from *A fumigatus* strain, AF293, which we previously showed lacked protease activity when grown in minimal culture media.^56^ With this in mind, we suggest that *A fumigatus*‐derived proteases may, in part, be involved in germinating spore‐mediated ET‐1 production but *A fumigatus*‐derived soluble factors other than proteases may also be driving ET‐1 production in the culture filtrate studies.

Secreted components in culture filtrate such as carbohydrate moieties and/or toxins may be driving induction of ET‐1 via activation of pattern recognition receptors such as Dectin‐1. For instance, in a similar allergic model of chronic lung exposure to live *A fumigatus* conidia, β‐glucan recognition via Dectin‐1 resulted in the induction of multiple proallergic and pro‐inflammatory mediators.^57^ In addition, gliotoxins and other metabolic by‐products are important *A fumigatus* virulence factors known to interfere with epithelial integrity^58^ and trigger the release of pro‐inflammatory mediators^53^ and possibly pro‐fibrogenic factors such as ET‐1. In vivo, where epithelial‐fibroblast crosstalk occurs, apoptosis of BECs may contribute fibroblast activation and fibrosis.^59^ We did not notice overt denuding of the epithelium in mice treated with *A fumigatus;* however, it is possible that *A fumigatus*‐induced ET‐1 and ultimately fibrosis are first initiated by transient apoptosis. Interestingly, gliotoxins have been shown to accentuate ovalbumin‐induced airway inflammation, Th2 sensitization and airway remodelling in a murine model.^60^ Of note, mechanical stress also induces the selective production of ET‐1 by bronchial epithelial cells in culture^61^ and ET‐1 was found to decrease bronchial epithelial cell proliferation and migration in vitro.^51^ Therefore, loss of bronchial epithelial cell integrity may induce ET‐1 production leading to subepithelial fibrosis and impaired epithelial repair. Lastly, TNF‐α is a known inducer of ET‐1 and this mediators has been shown to be up‐regulated in transformed human airway cells on exposure to germinating *A fumigatus* spores.^53^ Whether ET‐1 is induced indirectly via an early induction of TNF‐α in BECs exposed to *A fumigatus* may be another possible mechanism which requires further investigation.

It may seem surprising that there was no observable increase in expression of TGF‐β1 and TGF‐β2 and periostin when they are known to be associated with fibrotic response in multiple organs. It is plausible that the timing of analysis was a limitation, and a later time‐point may have shown an increased expression. However, evidence suggests that TGF‐β was not up‐regulated in mice exposed to *A fumigatus* spores unless they were pre‐sensitized by fungal extract intra‐peritoneally and subcutaneously.^62^ Furthermore, periostin does not appear to be essential for *A fumigatus*‐induced subepithelial fibrosis as mice deficient in periostin demonstrated the same extent of airway remodelling as wild‐type mice.^32^

Although our studies indicate an important role for ET‐1 in the aetiology of airway disease, there are several experimental limitations. In the current study, we used cultures of healthy human epithelial cells. Asthmatic nasal and bronchial epithelial cells are reported to produce heightened levels of the growth factors associated with fibrosis (TGF‐β2, periostin and VEGF) at baseline and in response to IL4/13 compared with healthy cells.^63^ Furthermore, ET‐1 release was found to be higher from unstimulated asthmatic epithelial cells compared to control cells.^64^ With these studies in mind, it is likely that if we had used asthmatic epithelial cultures, we may have observed an even greater amplitude ET‐1 production in response to *A fumigatus*. Furthermore, submerged alveolar epithelial cultures showed a dampened inflammatory response compared to those at air‐liquid interface (ALI) following an oxidative stress response with zinc oxide nanoparticles.^65^ Therefore, BECs grown at ALI are likely to have shown an even greater induction of ET‐1. The mouse models used in the current study, also fast‐track the allergic phenotype and recapitulate allergic features that may be comparable to some aspects of the disease, but by no means represent the complexity of asthma in people. Heightened exposure to fungal allergens may occur in asthma and other lung pathologies where pre‐existing mucus hypersecretion or cavitation provides the ideal environment for *A fumigatus* spores to thrive and avoid being cleared. Indeed, it is reported that 60%‐80% of asthmatics with fungal sensitization have *A fumigatus* present in sputum, suggesting that such people are continuously exposed to *A fumigatus‐*derived products at high concentrations over a long period of time.^6^

Herein, we have demonstrated for the first time that *A fumigatus* caused a robust up‐regulation of ET‐1 by bronchial epithelial cells and in murine lung. Antagonism of ET_A_ caused a profound decrease in inflammation and subepithelial fibrosis, highlighting the therapeutic potential for targeting ET‐1 in fungal‐sensitized asthma. Whether also blocking ET_B_ with a dual antagonist would have produced an even greater effect is not known. Although other studies have shown that antagonism of ET_B_ using BQ‐788 did not inhibit differentiation of fibrocytes into myofibroblasts^48^ and failed to influence airway inflammation^43^ suggesting that ET_B_ may not play a major role in ET‐1‐induced airway pathology. Of note, a small clinical trial using the dual receptor antagonist, bosentan, to treat people with asthma showed no improvement in the symptoms assessed^66^. However, this trial was limited by not reporting the specific allergic sensitization of participants and the fact that bosentan inhibits both ET‐1 receptors. Further studies assessing the efficacy of selective ET_A_ antagonism, specifically in *A fumigatus*‐sensitized asthma may be warranted.

## CONFLICT OF INTEREST

The authors declare no conflict of interest.
