# Supplementary figures and images for: Endothelin‐1 mediates Aspergillus fumigatus‐induced airway inflammation and remodelling
Source: Clin Exp Allergy. 2019 Mar 18;49(6):861–73. doi: 10.1111/cea.13367 (PMC6563189; doi:10.1111/cea.13367)

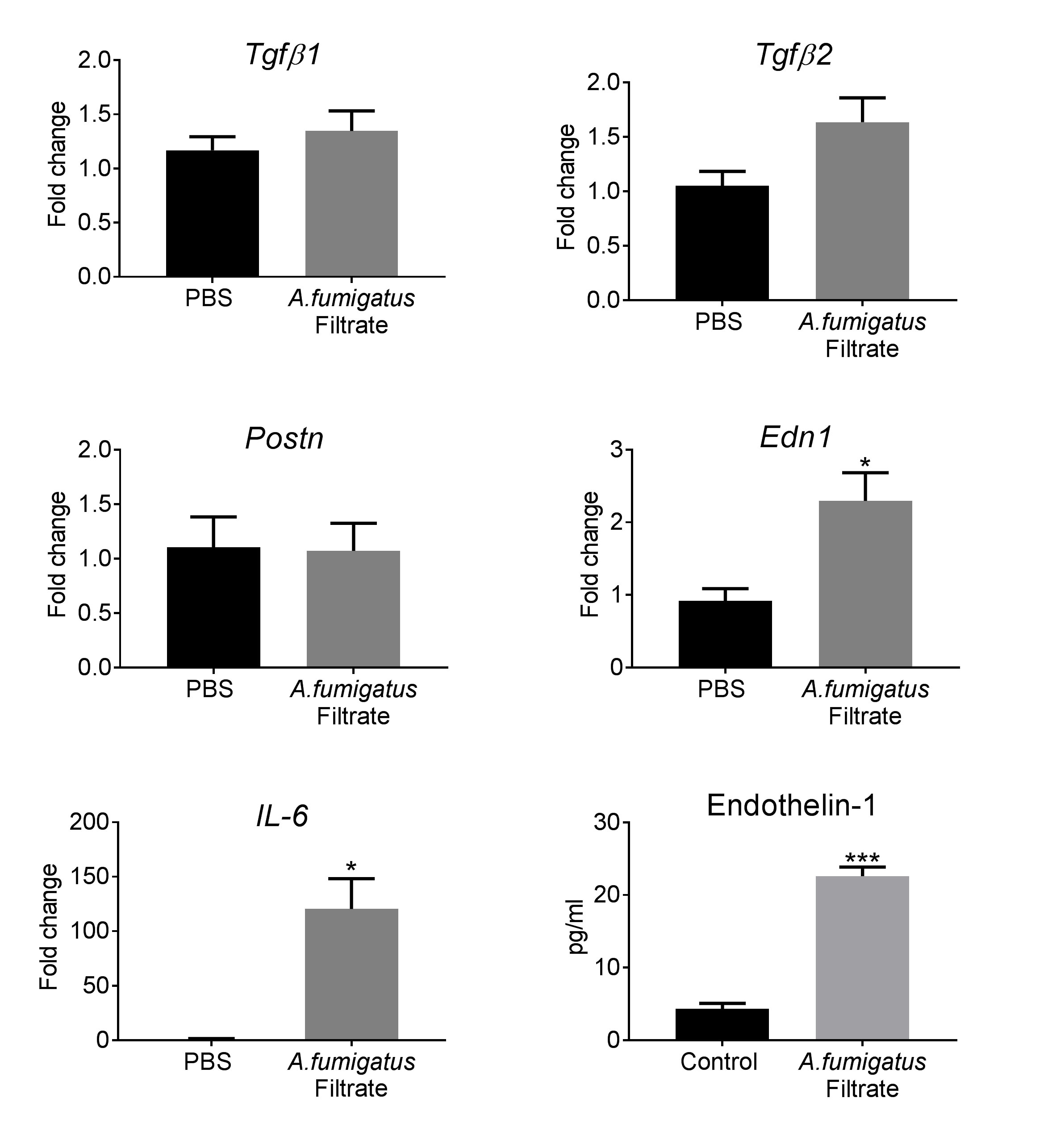

Supplement: Supplementary file 1 [file CEA-49-861-s001.jpg]

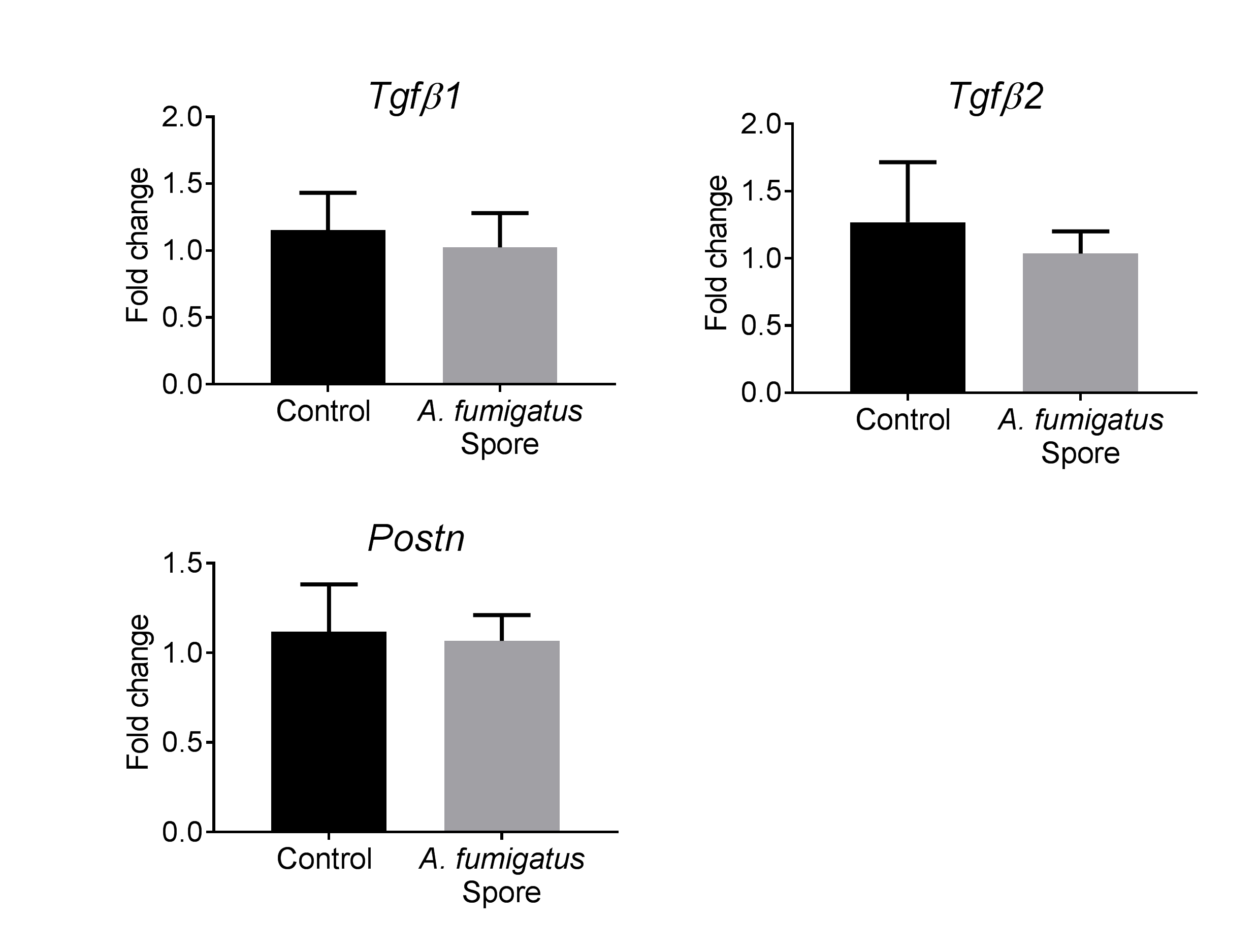

Supplement: Supplementary file 2 [file CEA-49-861-s002.jpg]

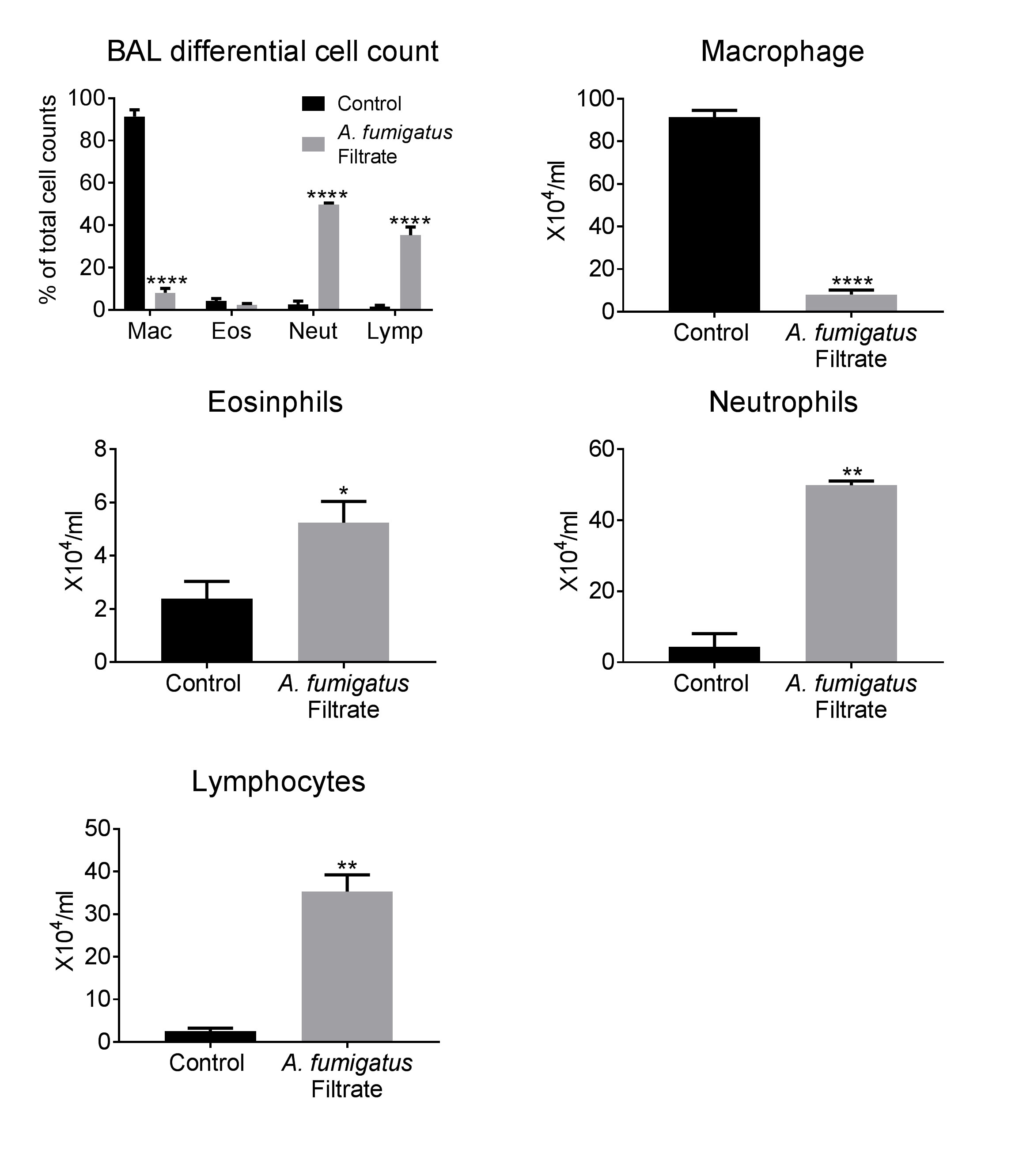

Supplement: Supplementary file 3 [file CEA-49-861-s003.jpg]

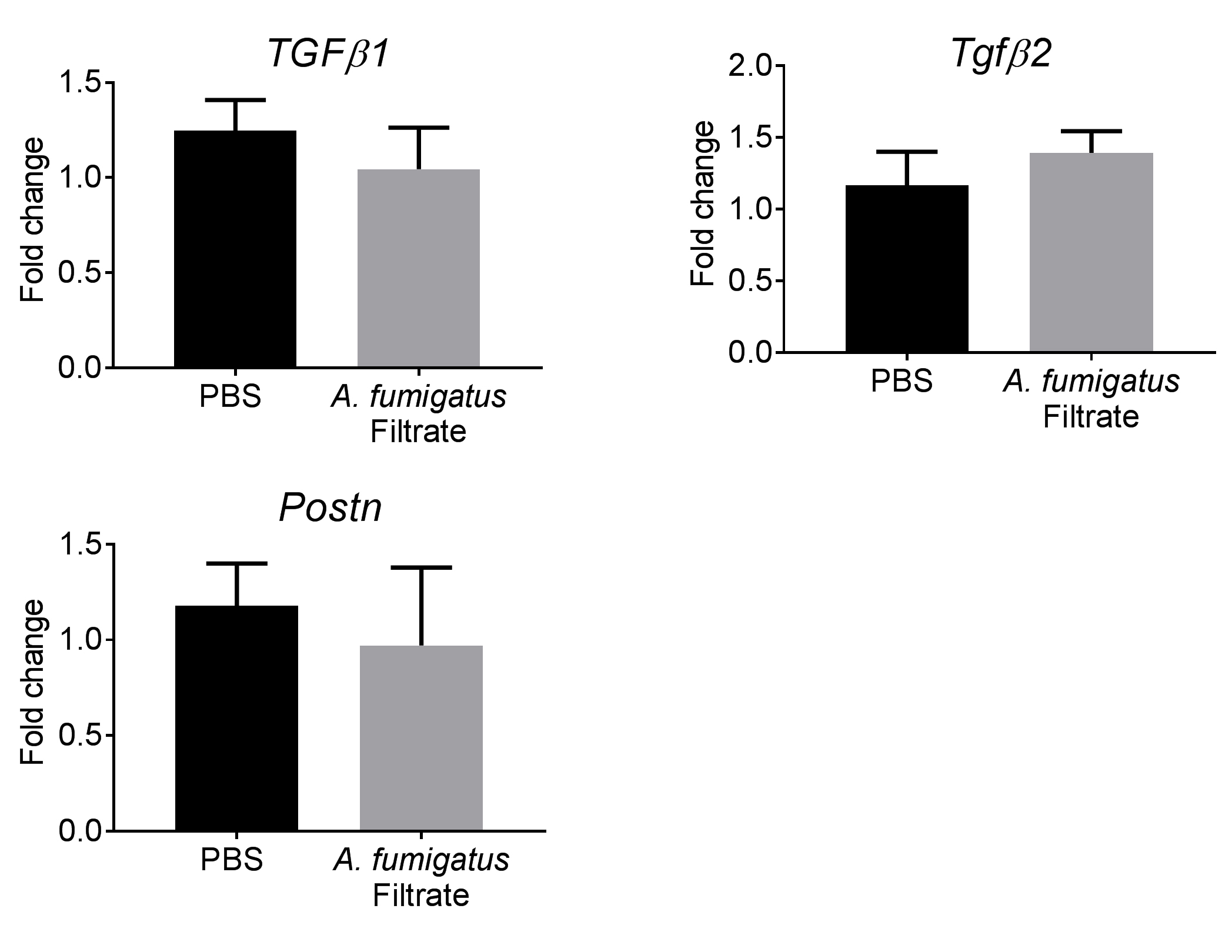

Supplement: Supplementary file 4 [file CEA-49-861-s004.jpg]

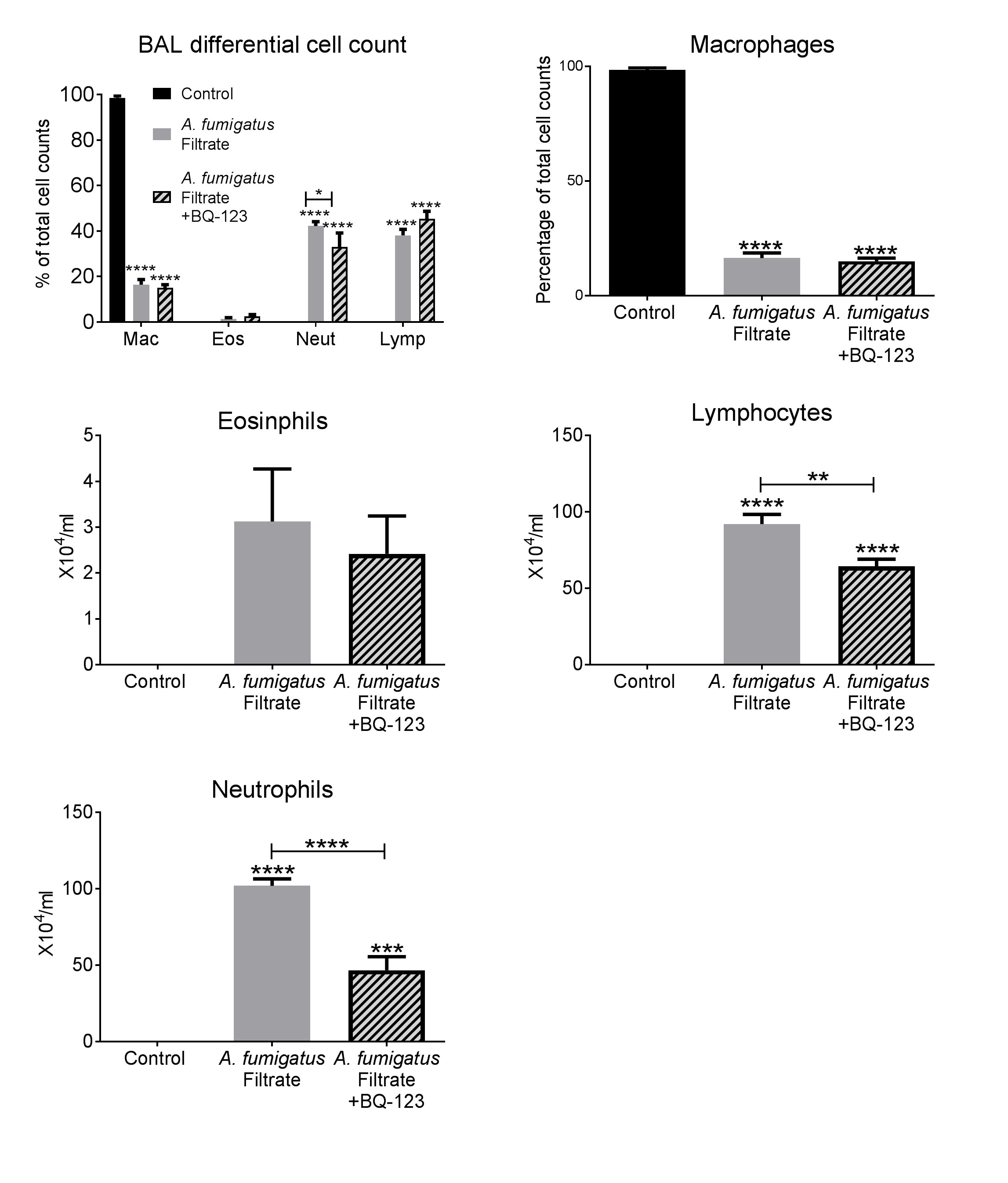

Supplement: Supplementary file 5 [file CEA-49-861-s005.jpg]
