# Supplementary material for: Endothelin‐1 mediates Aspergillus fumigatus‐induced airway inflammation and remodelling
Source: Clin Exp Allergy. 2019 Mar 18;49(6):861–73. doi: 10.1111/cea.13367 (PMC6563189; doi:10.1111/cea.13367)
Supplement: Supplementary file 6 [file CEA-49-861-s006.docx]

**Supplementary methods**

**Processing in vivo samples**

Blood and Bronchoalveolar lavage fluid (BALF) were spun at 12, 000 rpm for 5 minutes and resultant supernatants collected for ELISA. Lavage cell pellets were Giemsa stained (Sigma-Aldrich, Poole, UK) for differential cell counts. Lung tissue was collected for qRT-PCR (Middle lobe), fixed overnight in 4 % paraformaldehyde for histology and immunostaining (left lobe) and the right superior lobe homogenised in Pierce® RIPA buffer (Thermofisher Scientific, Loughborough, UK) containing protease inhibitor cocktail (Roche Diagnostics Limited, West Sussex, UK), and supernatants used for ELISA. Cytokines and growth factors were assessed by ELISA in BALF and lung homogenate. ELISA for IL-4 and IL-5 (all DuoSet ELISAs from R&D, Abingdon, UK), IL-6 (Quantikine ELISA R&D, Abingdon, UK) and Endothelin-1 (Enzo Life Sciences, New York, USA) were all performed according to the manufacturer’s instructions. Murine IgE ELISA (BD Biosciences, Oxford, UK) was performed on blood serum according to manufacturer’s instructions.

**Primers used for human epithelial studies**

| **Gene** | **Forward Primer** | **Reverse Primer** |
| --- | --- | --- |
|  |  |  |
| Endothelin-1 | TGAGAATAGATGCCAATGTGCTA | GAACAGTCTTTTCCTTTCTTATGATT |
| TGFβ1 | CACTCCCACTCCCTCTCTC | GTCCCCTGTGCCTTGATG |
| TGFβ2 | TACGCCAAGGAGGTTTACAAA | TGAAGTAGGGTCTGTAGAAAGTG |
| Periostin | GCTGCCATCACATCGGACATCTTG | GCTCCTCCCATAATAGACTCAGAACA |

**Primers used for *in vivo* mouse studies**

| **Gene** | **Forward Primer** | **Reverse Primer** |
| --- | --- | --- |
|  |  |  |
| TGFβ1 | TGGACACACAGTACAGCAAG | GTAGTAGACGATGGGCAGTG |
| TGFβ2 | AAAAACATCAAAACAAAACAGGAAAAT | GCAGAGAGCAATACAGAGGAA |
| Endothelin-1 | GTTGCCTGTGGGTGACTAATC | AACGCTTCTGACTCGGACA |
| Periostin | TTCCTCTCCTGCCCTTATATGC | CCTGATCCCGACCCCTGAT |

For qRT-PCR, housekeeping genes were selected using the Primer Design (Southampton, UK) geNorm™ Selection Kit. For bronchial epithelial cell culture, RPL13 was used as house keeper, whilst for the *in vivo* studies GAPDH was used. All primers were designed by Primer Design (Southampton, UK) Data was analysed according to the ΔΔCT method.
